# Evolution of novel sensory organs in fish with legs

**DOI:** 10.1016/j.cub.2024.08.014

**Published:** 2024-09-26

**Authors:** Corey AH Allard, Amy L Herbert, Stephanie P Krueger, Qiaoyi Liang, Brittany Walsh, Andrew L Rhyne, Allex N Gourlay, Agnese Seminara, Maude W Baldwin, David L Kingsley, Nicholas W Bellono

**Affiliations:** 1 Department of Molecular and Cellular Biology, Harvard University; Cambridge, 02138, USA; 2 Department of Developmental Biology, Stanford University School of Medicine; Stanford, 94305, USA; 3 Max Planck Institute for Biological Intelligence; Seewiesen, 82319, Germany; 4 Roger Williams University; Bristol, 02809, USA; 5 Department of Civil, Chemical and Environmental Engineering, University of Genoa; Genoa, 16145, Italy; 6 Lead contact

## Abstract

How do animals evolve new traits? Sea robins are fish that possess specialized leg-like appendages used to “walk” along the seafloor. Here, we show that legs are *bona fide* sense organs that localize buried prey. Legs are covered in sensory papillae that receive dense innervation from touch-sensitive neurons, express noncanonical epithelial taste receptors, and mediate chemical sensitivity that drives predatory digging behavior. A combination of developmental analyses, crosses between species with and without papillae, and interspecies comparisons of sea robins from around the world demonstrate that papillae represent a key evolutionary innovation associated with behavioral niche expansion on the seafloor. These discoveries provide unique insight into how molecular, cellular, and tissue-scale adaptations integrate to produce novel organismic traits and behavior.

## eTOC blurb

Allard *et al.* exploit an unusual fish called the sea robin, which possesses six leg-like sensory appendages, to understand how novel traits arise and diversify to drive new behaviors.

## Graphical Abstract

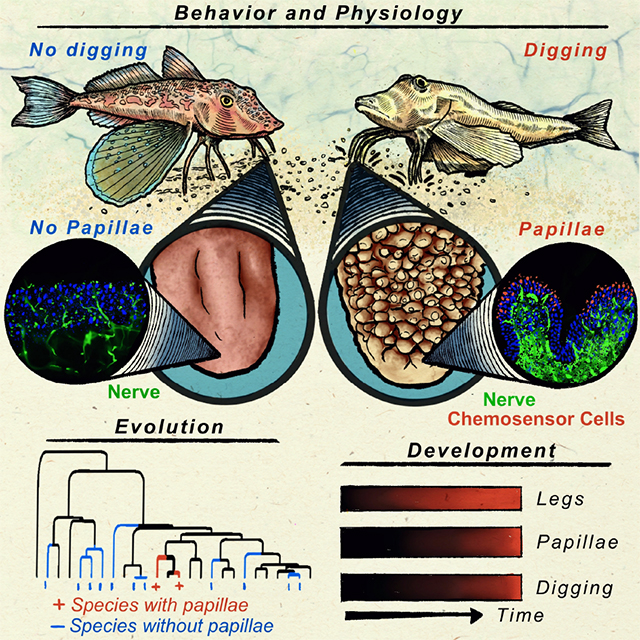

## Introduction:

The evolution and diversification of novel traits enables adaptation and expansion into new ecological niches. Yet, there are few examples that connect the molecular and developmental origins of a major trait gain with its physiological, behavioral, and ecological relevance. One remarkable example of trait gain is the “leg”-like appendage found within the family of fishes known as sea robins (Triglidae). Legs consist of six independently controlled, detached pectoral fin-rays used for “walking” and are thought to facilitate predation of food sources concealed beneath the seafloor ^1–4^. Indeed, sea robins are so adept at finding prey that other fishes follow them to steal otherwise undetectable potential food sources ^5^. By exploring this largely unstudied system, we made several serendipitous discoveries which define sea robin legs as novel sense organs that mediate species-specific functions suited to distinct behavioral niches.

## Results:

### Sea robins use sensory legs for digging

**Figure 1.**
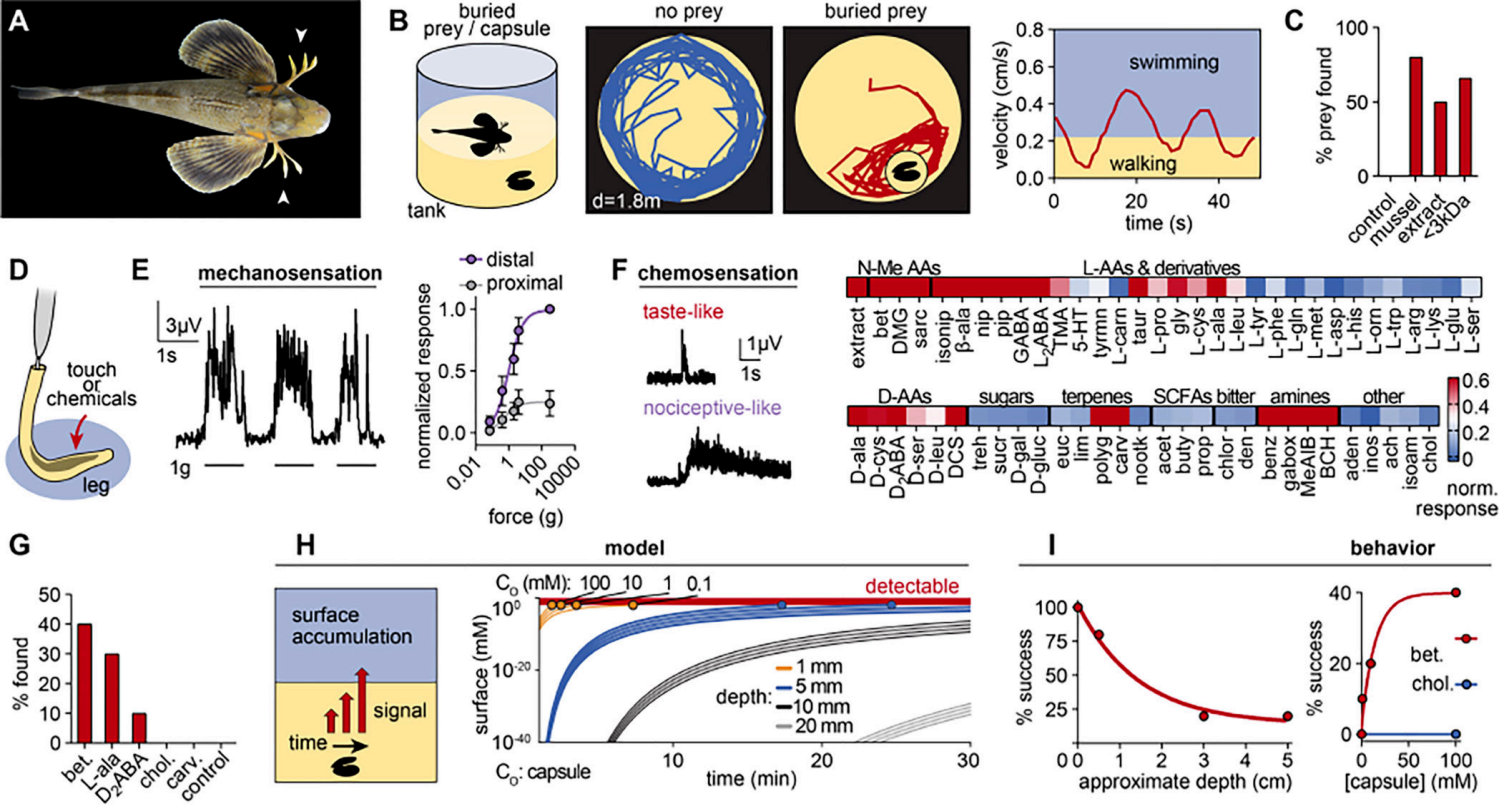
Sea robins are fish with sensory “legs” used to locate buried prey. (**A**) Sea robins (*Prionotus carolinus*) are fish with leg-like appendages (arrowheads). (**B**) Sea robins explored tanks with buried mussels by alternating between swimming (fast) and walking on sand (slow). Representative traces from 10 experiments. (**C**) Sea robins localized and uncovered buried mussels, or capsules containing mussel extract or <3kDa filtered extract, but not capsules containing control sea water (*n* = 10 trials each). (**D**) Schematic of electrophysiological recording from leg-specific spinal nerves. (**E**) Distal legs responded to mechanical stimulation from von Frey filaments of varying stiffness. *n* = 4 legs with 4–10 stimulations per filament per leg, p < 0.0001 for comparison of curve plateau by sum-of-square F-test, plateau = 0.8342 to 1.163 (distal) vs. 0.1959 to 0.3132 (proximal). Data represented as the mean ± s.e.m. (**F**) Legs responded to a broad range of chemicals including common tastants, marine osmolytes, and TRP channel agonists. Heatmap of relative responses from >6 legs. Responses to tastants (2 mM L-alanine) exhibited markedly faster kinetics compared to those elicited by TRP channel agonists (1 mM carvacrol). Recording representative of 6 recordings. Abbreviations: N-Me AAs: N-methylated amino acids, L-AAs: L-amino acids, D-AAs: D-amino acids, SCFAs: short chain fatty acids. (**G**) Sea robins found capsules containing solutions of single leg agonists but not inert chemicals (*n* = 10 experiments per chemical). (**H**) Model of chemical diffusion and surface availability in which shallow capsules of high concentrations produce detectable surface chemicals. (**I**) Sea robins found mussels buried at shallow depths and higher concentrations. *n* = 10 trials each. See also Figure S1 and Video S1.

Anecdotal stories of sea robins’ ability to find buried prey are common in the fishing community, but this behavior has not been examined experimentally. To test this ability, we developed a simple behavioral assay in which sea robins (*Prionotus carolinus*) were housed in a controlled tank with either mussels or capsules containing crude or filtered mussel extract buried in sand without visual cues (Figure 1A, B, Video S1). Sea robins alternated between short bouts of swimming and walking (Figure 1B) and appeared to “scratch” at the sand surface with their legs while walking, which we hypothesized represented sensory behavior. Indeed, sea robins regularly found all buried prey-related items but did not uncover control capsules containing sea water (Figure 1C), consistent with previous physiological evidence that the legs of some sea robins respond to chemical, tactile, and proprioceptive stimuli ^2,6^. To determine if leg sensation facilitates predatory digging behavior, we tested whether the leg nervous system transduces specific cues that evoke sensory behavior. Using electrophysiological recordings from spinal nerves that innervate legs, we found that distal legs were robustly mechanosensitive (Figure 1D, E). Furthermore, legs responded to prey extracts and common tastants for fish such as small non-polar L-amino acids ^7^, and other amine and imine molecules including D-amino acids, methylated amino acids, and GABA-related compounds (Figure 1F, Figure S1A). We also measured responses to compounds typically associated with nociception, such as TRP channel agonists, which elicited slower response kinetics compared with tastants (Figure 1F). Based on this physiological profile, we repeated behavioral experiments using capsules containing single molecules that evoked leg neural activity. Remarkably, sea robins successfully located capsules containing only single leg agonists such as L-alanine, betaine, or D_2_-aminobutyric acid (D_2_ABA), but not TRP channel agonists or inert compounds (Figure 1G). These results demonstrate that leg chemosensitivity for tastant-like molecules contributes to predatory behavior and suggest that legs could function as sensory organs.

How do sea robins use their legs to sense prey if they are covered by sand? Chemicals released by prey would need to diffuse through sand and accumulate at sufficient surface concentration for sensation. To understand the limitations of detection, we developed a mathematical model that describes diffusion in sand over a spatial scale that depends on time and the physicochemical qualities of stimuli emanating from buried prey sources. Using values consistent with our behavioral experiments, we found that stimulus sources must be concentrated and shallow to reach the surface without being significantly diluted (Figure 1H). To test this prediction, we asked if sea robins could find mussels buried at varied depths. As expected, the success rate of capture sharply decreased with depth (Figure 1I). Furthermore, sea robins were successful at finding capsules containing leg agonists ranging from 1 mM to 100 mM, corresponding with micromolar surface concentrations (Figure 1I). Thus, sea robin legs detect buried chemical sources using near-surface cues at micromolar concentrations, consistent with their benthic ecology and leg chemosensitivity.

### Digging sea robins have specialized legs

**Figure 2.**
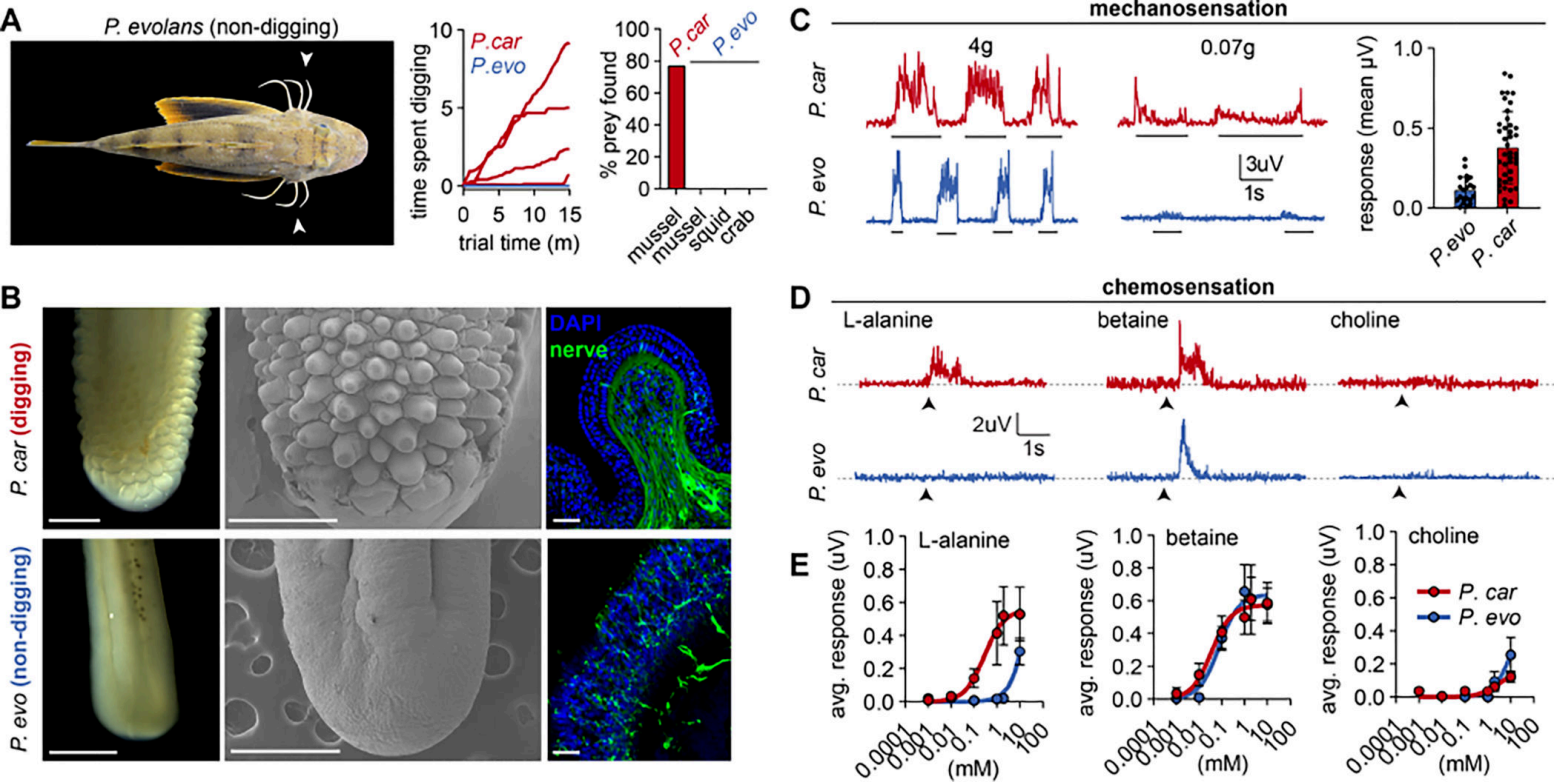
Legs of digging sea robins are specialized sensory organs. (**A**) (*left*) *Prionotus evolans*. (*right*) *P. carolinus* but not *P. evolans* exhibited digging behavior and found buried prey (*n* = 10 mussel trials per indicated prey per species, 4 representative traces of mussel experiments for both species). (**B**) Distal legs of digging *P. carolinus* were covered in papillae, which were absent in non-digging *P. evolans*. (*left*) brightfield, (*middle*) scanning electron microscopy, (*right*) immunofluorescence for neural antigen HNK-1 (green) revealed that leg papillae and ridges are densely innervated in *P. carolinus* and modestly in *P. evolans,* respectively. Scale bars (1 mm *left*, 500 μm *middle*, 25 μm *right*). (**C**) Legs of both species responded to strong mechanical stimulation (4g filament), but only *P. carolinus* responded to light mechanical stimulation (0.07g filament, *n* = 41 *P. carolinus*, 22 *P. evolans*, *p* <0.0001, t-test with Welch’s Correction) Data points represent mean signal amplitude averaged over poke duration. (**D**) Sensation of L-amino acids (2 mM) was unique to digging *P. carolinus*, while both species responded to betaine (2 mM) and showed little response to choline (2 mM). Representative nerve recordings of >5 legs. (**E**) Digging *P. carolinus* legs were ~100X more sensitive to L-amino acids than non-digging *P. evolans* (responses detected at 100 μM in *P. carolinus* vs 10 mM *P. evolans*). *n* = 4 legs. Data in **C** and **E** represented as mean ± s.e.m. See also Figure S1 and Figure S2.

During the course of behavioral experiments, we unexpectedly caught a second species of sea robin, *Prionotus evolans,* which also has legs (Figure 2A). Remarkably, we found that *P. evolans* failed to find buried mussels, squid, or crabs, despite consuming the same prey when visibly presented (Figure 2A). Based on these observations, we conclude that *P. carolinus* is a robust digging species while *P. evolans* is non-digging and uses its legs for locomotion and probing visible prey. Considering the prominent role of sensory legs in digging behavior, we wondered whether interspecies behavioral differences reflect leg specialization. To our surprise, we observed striking macroscopic differences in leg morphology between the two species. The distal legs of digging *P. carolinus* were shovel-shaped and covered in epithelial protrusions similar to papillae found on tongues and along the gastrointestinal tract of many animals (Figure 2B). In contrast, non-digging *P. evolans* legs were rod shaped, lacked papillae, and instead exhibited broad, shallow ridges in regions corresponding to papillae-covered surfaces in *P. carolinus* (Figure 2B). Based on these morphological differences, we hypothesized that papillae are specializations that facilitate digging behavior.

Leg papillae resemble sensory structures such as taste bud-containing oral papillae and mechanosensory papillae found on the facial regions of animals ranging from birds to star-nosed moles ^8^. However, leg papillae lacked markers for canonical chemosensory cell types such as those in taste buds, solitary chemosensory cells, and olfactory receptor neurons, which we could readily detect in other sea robin tissues (Figure S1B–F). Instead, the only putative sensory cells we observed within papillae were free nerve-endings that permeate the distal leg epithelium in both *P. carolinus* and *P. evolans*. Nerve endings were densely concentrated within individual papilla in *P. carolinus,* but distributed along the broad ridges of *P. evolans* legs ^6^ (Figure 2B). Consistent with established function of free nerve endings, the distal legs of both sea robin species were sensitive to strong mechanical stimuli. However, papillae-covered surfaces of *P. carolinus* exhibited enhanced touch sensitivity that could reflect an adaptation that allows digging species to differentiate among objects, textures, or motion in sand (Figure 2C, Figure S2B).

Chemical stimuli are required for sensory digging behavior, so we next wondered whether chemosensitivity differs between sea robin species. Whereas digging *P. carolinus* exhibited robust responses to common appetitive chemicals like L-amino acids, non-digging *P. evolans* legs were insensitive and instead only responded to a subset of chemicals, including nociceptor-associated molecules (Figure 2D, Figure S1A). Remarkably, the legs of digging sea robins were ~100X more sensitive to L-amino acids than non-digging species, consistent with behavioral results and limitations of stimulus diffusion (Figure 2E). Thus, enhanced chemical sensitivity in *P. carolinus* legs is associated with the ability to localize buried prey.

### Molecular and cellular basis of sensory adaptation

**Figure 3.**
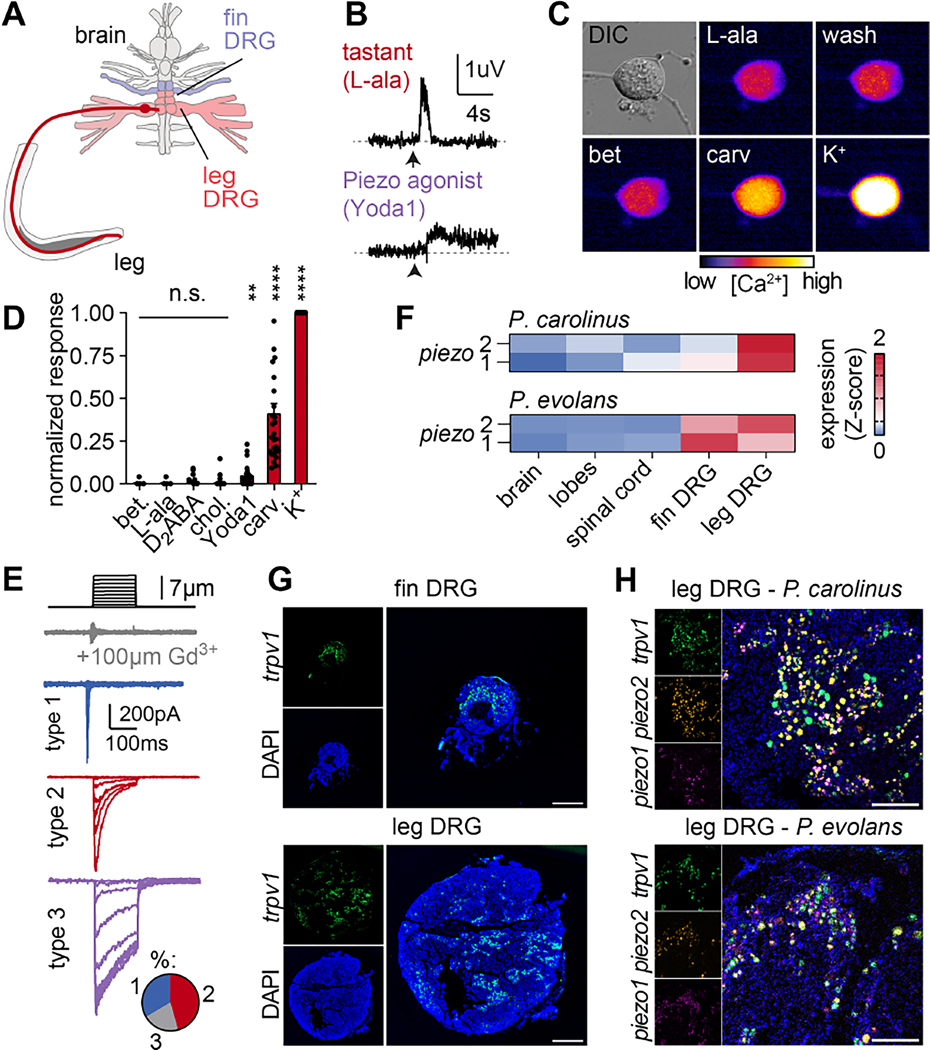
Sensory neurons mediate leg mechanosensation. (**A**) Diagram of sea robin leg neural architecture ^10^. (**B**) Responses to 2 mM L-alanine had faster kinetics compared to those elicited by the Piezo mechanoreceptor agonist Yoda1 (5 μM). Traces representative of 6 replicate recordings. (**C-D**) Cultured sensory neurons from *P. carolinus* leg ganglia responded to depolarizing K^+^ (70 mM), the TRP channel agonist carvacrol (100 μM), and weakly to the Piezo agonist Yoda1 (5 μM), but not to appetitive (10 mM betaine, L-alanine, L-proline, D_2_ABA) or control chemicals (choline 10 mM) (*n* = 28 cells, RM one-way ANOVA with Geisser-Greenhouse correction and Dunnet’s multiple comparison test to mean of Choline, $p_{\text{Adj}}=0.6730$ betaine, 0.6913 L-ala, 0.7702 D_2_ABA, 0.001, Yoda1, <0.0001 carvacrol, <0.0001 K^+^). Data represented as mean ± s.e.m. (**E**) Sensory neurons were mechanosensitive and could be separated into three functional populations (*n* = 26, 8 type 1 with fast desensitization, 11 type 2 with intermediate desensitization, and 5 type 3 with slow desensitization). (**F**) *piezo* mechanoreceptor mRNA transcripts were enriched in leg ganglia of both species. Scale: z-scaled normalized counts. (**G-H**) Leg-specific spinal ganglia were enlarged relative to fin ganglia and possessed an expanded population of *trpv1*-positive sensory neurons (*P. carolinus*, *trpv1* in green, DAPI in blue, scale bar 500 μm). Large populations of ganglia sensory neurons expressed mechanosensitive *piezo1* (magenta) and *piezo2* (orange) ion channels (DAPI in blue, scale bar = 200 μm). Images representative of 3 animals per species. See also Figure S2

What are the molecular and cellular mechanisms of enhanced sensation in digging sea robins? Free nerve endings are typically associated with mechanosensation and chemesthesis (thermal, nociceptive, and tactile sensations) ^9^, but not the detection of appetitive stimuli like the amino acids that elicit digging behavior. Accordingly, chemicals associated with nociception and the Piezo mechanoreceptor agonist Yoda1 elicited leg nerve responses with slow, sustained kinetics, while appetitive chemical agonists including the common tastant L-alanine elicited fast, transient responses, suggesting different mechanisms of sensation or transduction (Figure 3B, Figure 1H). We therefore sought to identify cell types associated with each sensory modality. Early retrograde tracing of nerve endings in legs demonstrated that their neuronal cell bodies reside in dramatically enlarged spinal dorsal root ganglia (DRG), which connect to six novel spinal cord lobes that are specific to each leg ^10^ (Figure 3A). These enormous ganglia are many times larger than any other of the sea robin’s spinal ganglia, suggesting that the evolution of legs as a novel sensory organ involved massive expansion in the population of sensory neurons. To characterize cellular properties, we dissociated and cultured functional sensory neurons from the specialized ganglia of digging *P. carolinus* (Figure S2). Whereas isolated neurons responded to Yoda1, the nociceptive TRP channel agonist carvacrol, and depolarizing high-extracellular potassium, we did not measure responses to appetitive leg agonists such as L-alanine (Figure 3C, D). Furthermore, most neurons exhibited mechanical stimulus-induced currents that varied in kinetics, suggesting a diversity of mechanoreceptor subtypes that is indicative of specialized somatosensory function (Figure 3E). Thus, we conclude that sensory ganglia neurons mediate leg responses to touch and nociceptor-associated molecules but do not detect appetitive chemicals.

We next considered the molecular basis of leg sensation. Consistent with the mechanosensitivity of legs and the sensory neurons that innervate them, leg ganglia from both species exhibited enriched expression of the mechanosensitive ion channels *piezo1* and *piezo2* but did not express known chemoreceptors for appetitive molecules (Figure 3F). Both *piezo* channels colocalized with the sensory neuron marker *trpv1* (Figure 3G, H) and *piezo1* transcripts were particularly enriched in neurons that innervate legs compared with those from pectoral fins (Figure 3G, H, Figure S2), suggesting that *piezo1* may serve an unappreciated role in environmental sensation in fishes ^11,12^. Considering that the leg-specific, enlarged dorsal root ganglia of both species contain sensory neurons expressing similar mechanosensitive ion channels, we conclude that mechanosensation across species shares a common molecular and cellular basis and suggest tissue-level adaptions (papillae versus ridges) underly species-specific mechanosensory properties.

**Figure 4.**
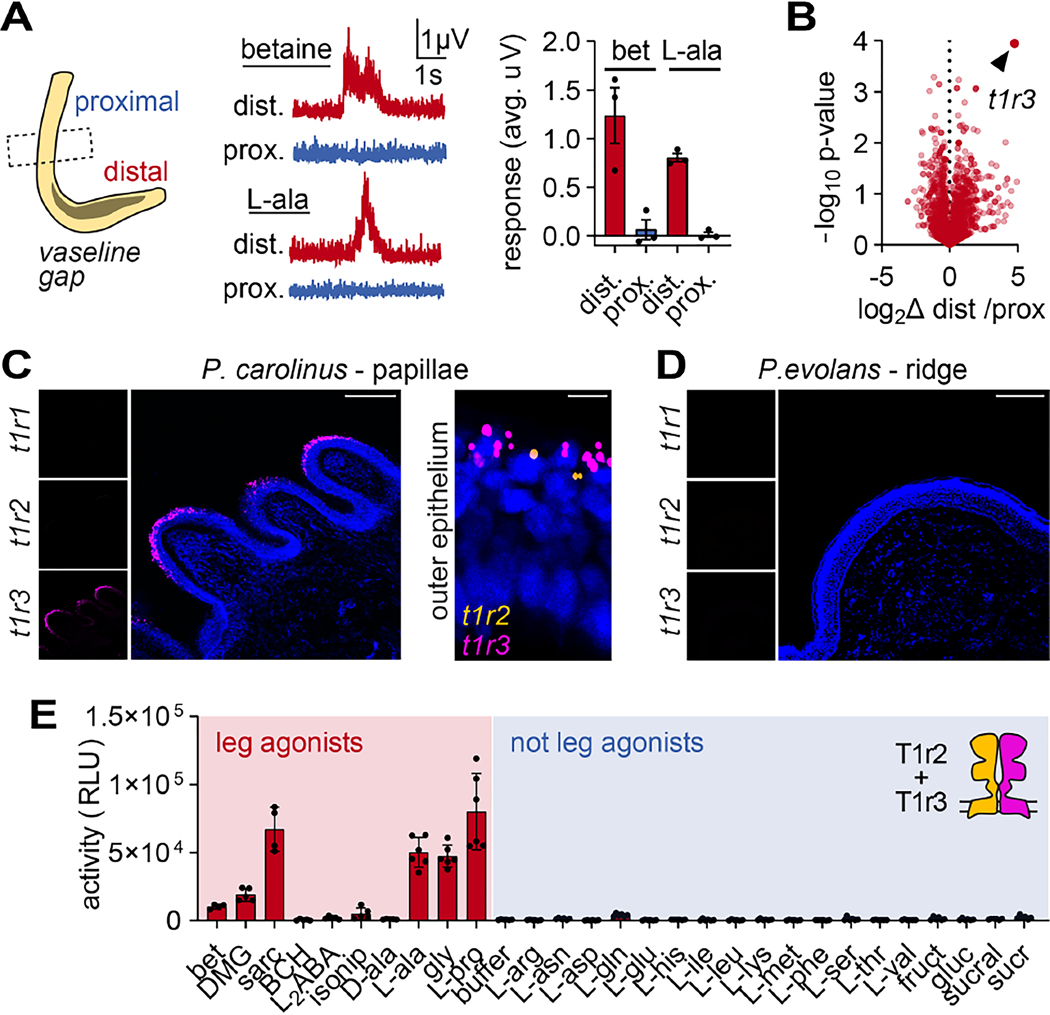
Taste receptors in papillae mediate leg chemosensation. (**A**) Only the papillae-covered distal leg, but not proximal leg surface of digging *P. carolinus* responded to appetitive chemicals (2 mM betaine or L-alanine, n = 3, *p* < 0.0180 betaine, *p* < 0.0001 L-ala, t-test with Welch’s correction). (**B**) The taste receptor *t1r3* (arrowhead) was the most enriched receptor in the chemosensitive distal leg epithelium. (**C-D**) *t1r3* and *t1r2* were expressed in surface epithelial cells of papillae in digging *P. carolinus*, but absent from non-digging *P. evolans*, visualized by *in situ* hybridization (scale bars = 100 μm for *left, right*, 10 μm for *middle*, images representative of 3 animals per species). (**E**) T1r2/T1r3 heterodimers (from *P. carolinus)* responded to the L-amino acids sensed by digging *P. carolinus* but not non-digging *P. evolans* (*n* = 6). RLU = Relative Light Units. Data in **A** and **E** represented as mean ± s.e.m. See also Figure S3.

If sensory neurons are not responsible for appetitive chemosensation, we wondered if this modality could be mediated by noncanonical sensory cells within the leg epithelium. To determine whether specific leg regions are specialized for chemosensation, we used a split-recording chamber and found that only the papillae-covered distal leg was responsive to appetitive chemicals (Figure 4A). We then exploited this anatomical specialization to identify candidate chemoreceptors using comparative transcriptomics from distal versus proximal legs. Strikingly, the taste receptor *t1r3* was the single most-upregulated receptor in the distal leg epithelium of digging *P. carolinus* (Figure 4B). At the cellular level, we observed robust and specific epithelial localization of *t1r3* mRNA within the tips of papillae, which specialize sensory leg function of digging sea robins (Figure 4C). T1r3 is canonically expressed in the specialized receptor cells of oral tastebuds, where it forms obligate heterodimers with T1r1 or T1r2 to mediate sensation ^7,13–15^ (Figure S3A). Indeed, we also detected expression of *t1r2* in papillae epithelial cells, albeit at lower levels, suggesting that functional heteromeric taste receptors could mediate leg chemosensitivity (Figure 4C). In contrast, we did not detect taste receptor expression in the legs of non-digging *P. evolans*, consistent with insensitivity to most common tastants (Figure 4D). To determine whether the selectivity of sea robin taste receptors matches leg physiology and behavior, we heterologously expressed *P. carolinus* receptors in HEK293T cells and measured responses to chemical agonists. Remarkably, we found that co-expression of T1r2 and T1r3 mediated robust responses to the same L-amino acids that distinguish digging *P. carolinus* chemosensitivity from that of non-digging *P. evolans* (Figure 4E, Figure S3B,C) ^16,17^. Considering these results, chemosensation appears to have arisen through the evolution of a novel chemosensory epithelial cell type rather than sensory neurons, which play a conserved role in mechanosensation. Thus, papillae are molecularly defined polymodal sensory structures that specialize sea robin legs to support digging behavior.

### Leg sensory organs as a major trait gain that enables novel behavior

**Figure 5.**
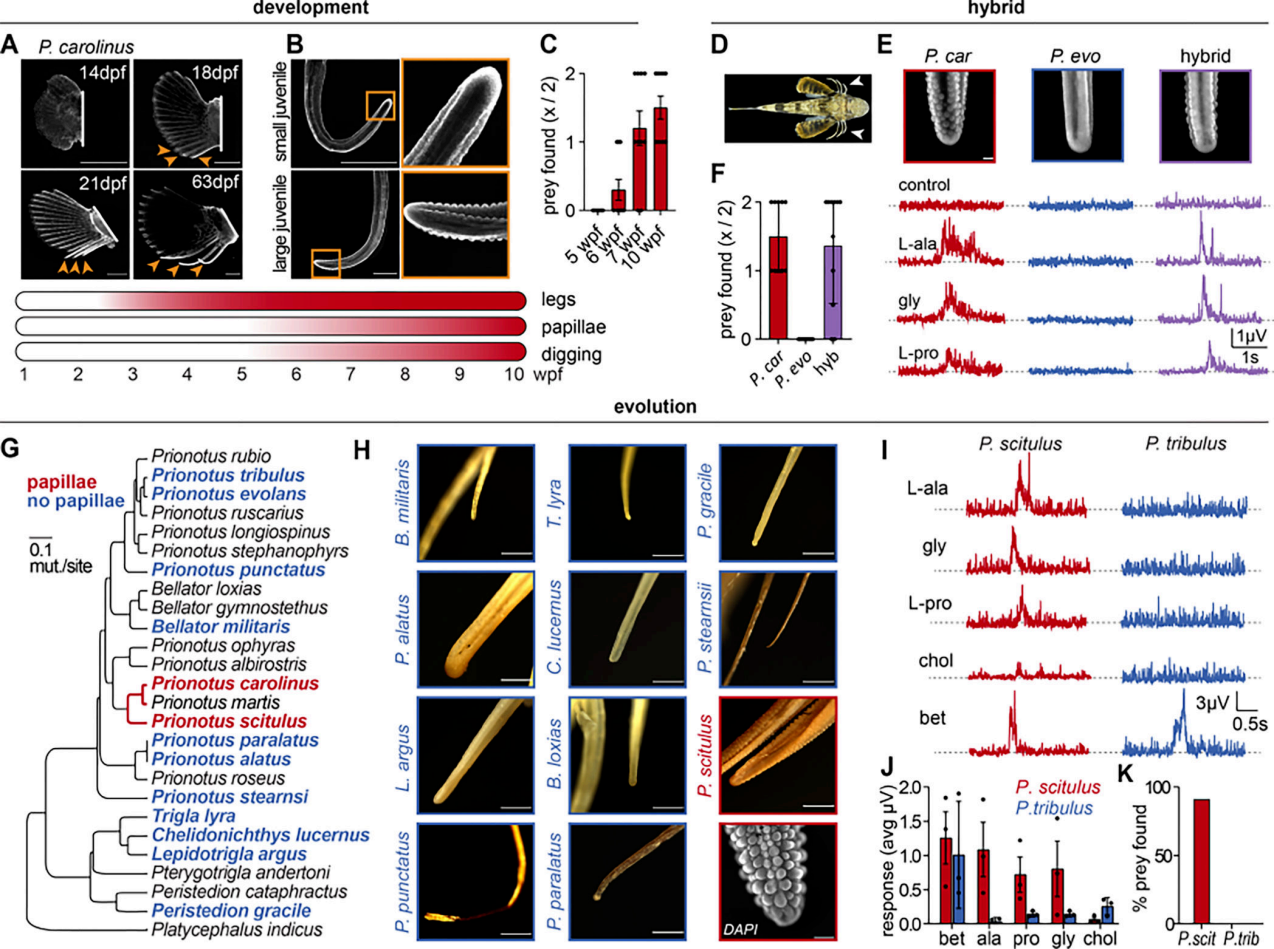
Papillae are a major gained trait and facilitate predatory digging behavior. (**A**) *P. carolinus* legs formed weeks after hatching (stained with DAPI, scale bars = 1 mm). dpf = days post fertilization. (**B**) Legs of larval sea robins initially lacked papillae, which later formed in larger juveniles (scale bars = 1 mm). wpf = weeks post fertilization. (**C**) Onset of digging behavior correlated with papillae formation in developing *P. carolinus* sea robins (*n* = 10 trials per age group, key below indicates approximate timeline with shading beginning at first observation of trait, ANOVA with Tukey’s post-hoc test: 5 vs 6 wk. n.s., 5 vs 7 wk. p < 0.0001, 5 vs 10 wk. p < 0.0001). (**D**) Dorsal view of an F1 hybrid sea robin produced from crossing female *P. evolans* and male *P. carolinus.* (**E**) Hybrid progeny have leg papillae (*top*, scale bar = 250μm) and responded to appetitive chemicals similar to their digging parent. Representative image of *n* = 4. (**F**) Hybrid sea robins exhibited robust digging behavior to find prey, similar to parental *P. carolinus* that also have leg papillae (*n* = 10, *p* < 0.0001 hybrid or *P. carolinus* vs *P. evolans* by ANOVA with Tukey’s post-hoc test). (**G**) Species with leg papillae (red) represent a restricted clade of the sea robin phylogenetic tree ^21^. Species in blue lacked papillae, and species in black were not analyzed. (**H**) Leg morphology of museum specimens and DAPI stained papillae in *P. scitulus* (scale bar = 200 μm). (**I-J**) *P. scitulus*, which has leg papillae, responded to appetitive L-amino acids, while *P. tribulus*, which lacks papillae, was insensitive (*n* = 4 recordings per chemical per species). (**K**) Papillae-expressing and chemosensitive *P. scitulus* exhibited robust digging behavior to find prey, while *P. tribulus* did not dig or find prey (*n* = 10 trials). Data in **C**, **F**, **J** represented as mean ± s.e.m. See also Figure S4.

To further interrogate the role of papillae in digging behavior we examined their function in three contexts: (1) sea robin ontogeny; (2) interspecies hybrid animals; and (3) across the diverse phylogenetic tree of sea robins. Larval sea robins hatch without legs and are initially pelagic. Then, legs separate from pectoral fins during the first three weeks of development as the animals settle to the sea floor ^18–20^ (Figure 5A). While observing this transition in the lab, we discovered that *P. carolinus* lacked papillae during the first five weeks of development (Figure 5B). Strikingly, *P. carolinus* began to localize and dig up prey at a stage coinciding with papillae formation, suggesting that the presence of papillae facilitates digging behavior (Figure 5C). Next, we considered the pattern of inheritance in crosses of hybrid animals with distinct parental traits. Crosses of digging *P. carolinus* males with non-digging *P. evolans* females produced viable offspring that could be raised through the stage of leg-separation to adult morphology (Figure 5D). Hybrid animals developed legs that possess papillae which closely resemble the digging parent (Figure 5E). Consistent with a critical role in sensation, papillae in hybrid sea robins correlated with enhanced chemosensitivity and sensory digging behavior (Fig 5E–F). In a companion paper, we leveraged these hybrid animals to examine gene regulatory mechanisms underlying species-specific trait inheritance and found that a combination of *cis*- and *trans*- regulatory elements drive trait inheritance in hybrids (*Herbert, et al*). These results collectively support the hypothesis that papillae are required for sensation and predatory digging.

Finally, we leveraged the biodiversity of sea robin species from around the world to ask whether sensory specialization reflects the ancestral condition or is a recent innovation. We first used museum specimens to examine the leg morphology from a wide range of sea robin species (Figure S4). Surprisingly, we observed leg papillae in only a small group of sea robins comprising the closest relatives of digging *P. carolinus* (Figure 5G, *red text*). In contrast, the legs of many species exhibited a stick-like morphology similar to fin rays, suggesting these structures may function as simple locomotory appendages that are ancestral to more specialized sensory legs (Figure 5H). Guided by this analysis, we obtained and examined the physiology and behavior of two additional species, one with papillae (*Prionotus scitulus*) and one lacking papillae (*Prionotus tribulus*). Only *P. scitulus* exhibited chemosensory specialization and digging behavior, consistent with its leg morphology and close evolutionary proximity to *P. carolinus* (Figure 5I–K). Therefore, leg papillae are sensory specializations unique to a specific clade of sea robin species and represent a novel trait gain.

We conclude that sensory digging behavior is restricted to a small lineage of specialist fishes that have diversified legs as novel sense organs, evolving unique molecular, morphological, and functional properties in sensory end-organs. We hypothesize that sea robins initially developed fin ray-like legs for locomotion. Ancestral organs then evolved limited sensory capability to facilitate manipulation of the visible substrate in search of food. Finally, evolution of sensory papillae further specialized legs to localize and uncover buried prey. Thus, we propose that sensory papillae represent a striking example of a major trait gain within a specific lineage that allows organismal expansion into a new behavioral niche. Indeed, our companion study demonstrates that legs are patterned by developmental programs which share features with other animal forelimbs and additionally contribute to the specialization of papillae as sensory structures required for digging behavior (*Herbert, et al*). Future work will leverage the remarkable biodiversity of sea robins to understand the genetic basis of novel trait formation and diversification in vertebrates by focusing on molecular mechanisms of fin ray separation, adaptation of the nervous system, and acquisition of sensory properties. Thus, our work represents a basis for understanding how novel traits evolve to facilitate the lifestyles of diverse organisms across distinct ecologies.

## Star Methods

### Resource Availability

#### Lead contact

Further information and requests for resources and reagents should be directed to and will be fulfilled by the lead contact, Corey Allard (coreyallard@fas.harvard.edu).

#### Materials availability

Plasmids generated in this study are available upon request. No other unique reagents were generated in this study.

#### Data and code availability

Sequencing data generated from this study are available at the NCBI Sequence Read Archive under BioProject: PRJNA1141128, accession numbers SRR30014073-SRR30014098. Any additional information required to reanalyze the data reported in this paper is available from the lead contact upon request.

### Experimental Model and Study Participant Details

#### Cell Culture

HEK293 cells (ATCC, authenticated and validated as negative for mycoplasma by vendor) were cultured in Dulbecco’s Modified Eagle Medium (DMEM) (Gibco) supplemented with 10% fetal calf serum (FBS) (Gibco) and 50 I.U./mL penicillin and 50 μg/mL streptomycin (Gibco) at 37 °C, 5% CO_2_ using standard techniques.

#### Sea robins

Adult wild-caught sea robins were provided by the Marine Biological Laboratory, Woods Hole, MA, (Prionotus carolinus and Prionotus evolans) or Gulf Marine Spcimens (Prionotus scitulus and Prionotus tribulus) and kept group-housed on a 12 hr light/dark cycle in natural sea water and fed. Larval sea robins were raised in Modular Larval Rearing System (MoLaRS) and fed daily with microalgae or brine shrimp, and kept on a 12 hr light/dark cycle in natural sea water. Experiments were performed using a mix of male and female animals as available, for which we observed no clear differences. Where required for experiments, animals were euthanized by immersion in Tricaine-S (Syndel) until 10 minutes past cessation of opercular movements. Animal protocols were approved by the Harvard University and Roger Williams University Animal Care and Use Committees (protocols HU: ID 18–05-324–1, RWU: R19–07-09).

### Method details

#### Behavior experiments

##### Adult behavior:

Sea robins were placed in a 1.5 m diameter pool containing a volume of ~750 L (or 200 gallons) of sea water and ~8 cm of sand. Experiments were filmed from above by a GoPro HERO 7 cameras (GoPro, Inc.) and evaluated using Windows Media Player (Microsoft). Fish were acclimated for 20 minutes in the behavior tank before experiments. Prey items were added to the tank at indicated depths just prior to experiment start using an opaque divider to prevent visualization of the bury site. Blue mussels (*Mytilus edulis*) were opened and halved along the hinge. Capsules were placed inside of a cleaned mussel shell to provide a physical object for capture. All behavior experiments were run for 30 minutes, and scored as negative if the mussel was not found within this time. Trials were scored as successful if prey items were excavated, or if a characteristic “head snap” behavior was noted at the site of buried prey, indicating prey was found. For experiments involving much smaller *P. scitulus* and *P. tribulus* species, smaller tanks and mussels were used (9 L, 23 × 34 cm).

##### Juvenile behavior:

Groups of juvenile sea robins (*P. carolinus*, *P. evolans*, and hybrids) were acquired from Roger Williams University and raised in seawater tanks. Prior to behavioral experiments, sand was thoroughly cleaned, and animals were not fed that morning. Behavioral experiments were then performed as for adults, except two mussels were added per tank. The mussel pieces changed position in the tank each time.

#### Molecular Biology

Complete cDNA sequences of taste receptors were obtained using a SMARTer 5’/3’ kit for RACE PCR to obtain 5’ sequence information missing from reference transcriptome (Takara, #634858). Briefly, RNA was extracted from oral and leg tissues using standard Trizol extraction, purified with Zymo Clean and Concentrator Kit (Zymo research #R1013), and diluted in 10–20 mL RNase free H2O without DNAase treatment. Taste receptor specific primers were used to amplify the 5’ end and PCR products were cloned as suggested by the company and verified by sequencing:

*t1r1* 5’ RACE primer:

5’- GATTACGCCAAGCTTGGCAGTGGTATTCATTTGTGGCCTCAGG

*t1r2* 3’ RACE primer:

5’- GATTACGCCAAGCTTCATGATCTTGCTTCTCTGTCTCTGCTGG

*t1r3* 5’ RACE primer:

5’- GATTACGCCAAGCTTTTAGTTCCCCGGCTCTGGCTGTGGCTGT

All constructs for functional assays were codon-optimized and synthesized by Genscript (Piscataway, NJ) into the pEAK10 expression vector.

#### Mathematical modeling

Chemical cues from a buried target spread mostly by diffusion because in sand water flow is largely suppressed. Qualitatively, chemicals diffuse over a spatial scale l that depends on the time t since it was released from the target $l\approx \sqrt{Dt}$, where D≈kT/(6πηr) is the chemical diffusivity and depends on the temperature and viscosity of water (T and η) and the size of the odorant molecule (r). Leg agonists are small molecules and their diffusivity in water at room temperature is typically $D\approx 10^{-10}$ to $10^{-9}m^{2}/s(D\approx 5\times 10^{-10}m^{2}/s$ for dopamine^22^ and fluorescein^23^ and closer to $D\approx 10^{-9}m^{2}/s$ for various aminoacids^24^). Thus, by the end of our experiments, the chemicals may only travel $\sqrt{Dt_{max}}\approx 1mm$ where $t_{max}=30^{\prime }$ is the duration of the experiment. This simple argument shows that only a thin ≈mm layer of sand in contact with the mussel is imbued with chemical signal, hence fish must touch sand close to the mussel in order to detect a signal.

In order to move beyond this qualitative argument, we focus on the betaine-capsule experiment for which we can predict the chemical concentration everywhere in space and time and compare it with leg sensitivity Betaine is a small amino acid derivative and we control its volume and concentration in the capsule ($c_{0}=0.1$ mM; 1 mM; 10 mM or 100 mM; V=1mL; thus the capsule contains $M=c_{0}V$ moles of betaine; we use $D\approx 10^{-9}m^{2}/s$ relevant for aminoacids^24^). Additionally, we know that the legs sense betaine above a threshold concentration $c^{*}=10$ to 100 μM (Figure 2E).

To complete the mathematical model, we need boundary conditions. The boundaries of the tank are irrelevant since they are much further than the diffusive length scale ≈1mm. The boundary condition at the water/sand interface depends on flow of water in the tank above sand. Since we do not control water flow in the experiments, we consider two opposite scenarios; the actual dynamics is somewhere in between these scenarios. If water is perfectly still, there is no boundary condition at the sand/water interface. A small portion of the capsule located at $r_{s}=\left(x_{s},y_{s},z_{S}\right)$ releases $dM=M\;dx_{S}dy_{S}dz_{S}$ moles of betaine and results in a molar concentration of betaine dc at any point in space and time^25^:

(1) $$dc(x,y,z,t)=\frac{dM}{8{\left(\pi Dt\right)}^{3/2}}e^{-r^{2}/(4Dt)}$$

where $r^{2}={\left(x-x_{s}\right)}^{2}+{\left(y-y_{S}\right)}^{2}+{\left(z-z_{S}\right)}^{2}$; the water/sand interface is located at z=0, water is at z<0 and sand is at z>0. Second, if water flows fast, betaine is greatly diluted away resulting in a Dirichlet boundary condition and using the method of images we obtain a modified prediction:

(2) $$dc(x,y,z,t)=\frac{dM}{8{\left(\pi Dt\right)}^{3/2}}\left(e^{-r^{2}/(4Dt)}-e^{-\rho^{2}/(4Dt)}\right)$$

where $\rho^{2}={\left(x-x_{S}\right)}^{2}+{\left(y-y_{S}\right)}^{2}+{\left(-z-z_{S}\right)}^{2}$. The total betaine concentration is obtained in both scenarios by summing the contribution dc from all portions of the capsule. Integrating over the volume V of the capsule (a prolate ellipsoid of dimensions 6.3 mm and 16 mm located at z=l) we obtain:

(3) c(x,y,z,t)=∫dc

We compute the integral in equation (3) numerically. The concentration of betaine in the topmost ≈0.5mm layer of sand which is presumably sampled by legs (Fig 1h): the two scenarios provide similar predictions hence we will ignore water flow for our current purposes. The conclusion from this calculation is that the concentration of betaine in sand is initially too small to be detected by the legs. After an initial transient, betaine can be detected on sand above the capsule. The duration of the initial transient depends on betaine concentration in the capsule, consistent with experiments in (Figure 1i) showing fish finds most efficiently the more concentrated capsules.

#### Electron microscopy

Electron microscopy was performed by the Harvard Medical School Electron Microscopy Core Facility according to standard protocols. Briefly:

##### Scanning electron microscopy:

Samples were fixed overnight in a mixture of 1.25% formaldehyde, 2.5% glutaraldehyde and 0.03% picric acid in 0.1 M Sodium cacodylate buffer, pH 7.4, washed with 0.1 M sodium cacodylate buffer, and post fixed with 1% osmium tetroxide in 0.1 M sodium cacodylate buffer for 2 hours. Tissues were then rinsed in ddH_2_0 and dehydrated through an ethanol series (30%, 50%, 70%, 95%, (2x)100%) for 15 min per solution. Dehydrated tissues were dried in an autosamdri-815 critical point dryer and mounted on aluminum stages with silver paint and coated with platinum (10 nm). The dried tissues were imaged on a Hitachi S-4700 Field Emission Scanning Electron Microscope (FE-SEM) at an accelerating voltage of 5kV.

##### Transmission electron microscopy:

Samples were fixed overnight in fixative solution (1.25% formaldehyde, 2.5 % glutaraldehyde, and 0.03% picric acid in 0.1 M sodium cacodylate buffer, pH 7.4), then washed with 0.1 M sodium cacodylate buffer and post-fixed with 1% osmium tetroxide/1.5% potassium ferrocyanide (in H_2_O) for 2 hours. Samples were then washed in a maleate buffer and post fixed in 1% uranyl acetate in maleate buffer for 1 hour. Tissues were rinsed in ddH_2_0 and dehydrated through an ethanol series (50%, 70%, 95%, (2x)100%) for 15 min per solution. Dehydrated tissues were put in propylene oxide for 5 min before they were infiltrated in epon mixed 1:1 with propylene oxide overnight at 4 °C. Samples were polymerized in a 60 °C oven in epon resin for 48 hours and then sectioned into 80 nm thin sections and imaged on a JEOL 1200EX Transmission Electron Microscope.

#### Transcriptomics

We generated tissue-specific *de novo* transcriptomes for *Prionotus carolinus* and *Prionotus evolans*. Tissues were dissected and stored frozen in RNAlater until use. RNA extraction, library preparation, and RNA sequencing were performed by the Harvard Bauer Core Facility using NovaSeq (2×250) platform or by Genewiz (Azenta) using a HiSeq (2×150 bp) platform. Adaptor trimming was performed using trimgalore^26^, and reference transcriptomes were assembled *de novo* using Trinity^27^. Open reading frames were identified using transdecoder^27^. Reads were pseudoaligned and transcript abundance was estimated using Kallisto^28^ and our novel transcriptome assemblies as a reference. Annotation was performed using Diamond^29^. Sequencing data generated from this study are available at the NCBI Sequence Read Archive under BioProject: PRJNA1141128, Accession numbers SRR30014073-SRR30014098.

#### Fluorescence In situ hybridization (RNAscope)

Tissues collected from recently euthanized specimens were immediately frozen in OCT and stored at −80°C until use. Blocks were trimmed and sectioned (18–50 μm) using a cryostat (Leica CM3050S). Probes were designed by ACD Inc. and the manufacturer-recommend protocol was followed as described for fresh frozen tissues. Pretreat 3 was used for 30min and fluorescent probes used included TSA-FITC, TSACy3 and TSA-Cy5 (Perkin Elmer #NEL744E001KT and #NEL754001KT), samples were mounted in ProLong Gold (Thermo Fisher #P36931) with DAPI, and imaged with a LSM 980 Confocal Microscope with Airyscan2 (Zeiss), LSM 900 Confocal Microscope (Zeiss), or AxioZoom v16 microscope. Images were processed using FIJI (NIH).

#### Primary neuron culture

Primary neuron culture was based on protocols established for zebrafish^30^ and optimized to improve cell survival as determined by cell density, neurite out-growth, and expression of voltage-gated conductances. Animals were euthanized and spinal ganglia corresponding to the legs and/or pectoral fin were dissected and placed in ice-cold HBS (HEPES buffered saline). Care was taken to remove blood from the tissue, as blood cells persist through the cell purification protocol. Ganglia were cut into small pieces and cells were dissociated by incubating ganglia pieces in enzyme solution (10 mg/mL collagenase IV (Worthington) in 1XHBS (HEPES buffered saline, Thermo)) for 1 hour at room temperature with gentle agitation. Enzyme solution was carefully removed and replaced with 10 mL culture media (L-15 media (Gibco #11415064) supplemented with 10% fetal calf serum (FBS) (Gibco) and 50 I.U./mL penicillin and 50 μg/mL streptomycin (Gibco), and 0.5 nM neurotrophin-3 (NT-3, Sigma #SRP6007)). Cells were released by trituration through fire-polished glass pipettes of decreasing diameter, until tissue was nearly completely broken up. Undissociated tissue was removed by passing cells through 100 μm nylon mesh filters (Corning). Debris and glial cells were removed by centrifuging cells over a 2 mL 15% bovine serum albumin (BSA, Sigma) solution in culture media at 250xg for 10 minutes at 4ºC. The entire supernatant was removed and the pellet containing enriched sensory neurons was resuspended in fresh 1 mL culture media. For plating, glass-bottomed Mattek dishes coated with poly-L-lysine were further coated in 1 mg/mL laminin in H_2_0 (Corning) for 1 hour, and rinsed 3X with sterile water. 100 μL cells were spotted into the center of each dish, allowed to adhere for several hours before addition of 1 mL of culture media. Cells were cultured in humidified chambers at 17ºC for 3–5 days before use in functional experiments.

#### Calcium imaging

Cultured neurons were washed in extracellular solution (in mM): 140 NaCl, 5 KCl, 10 HEPES, 2 CaCl2, 2 MgCl2, 10 D-galactose pH 7.4. and loaded with 5μM Fluo-8 AM (Abcam) and 0.01% Pluronic F-127 diluted in extracellular solution for 15min in the dark at room temperature, and washed 3X in extracellular solution before imaging. Imaging experiments were performed using a custom inverted wide-field fluorescence microscope running Metamorph imaging software. Perfusion experiments were performed using a SmartSquirt Micro-Perfusion system (Automate Scientific) pressurized to ~30 kPa. Stimuli were delivered for one minute followed by two minute wash intervals.

#### Museum Samples

Museums samples were staged in ethanol in glass dishes and legs were imaged using an AxioZoomV16 microscope equipped with a color camera (Zeiss). Images of entire animals were taken with a Cybershot digital camera (Sony).

#### Cell transfection

HEK293 cells were washed with Opti-MEM Reduced Serum Media (Gibco) and incubated with transfection mix containing 1 μg total of the indicated plasmid DNA and 3 μL Lipofectamine 3000 Transfection Reagent (Invitrogen) in Opti-MEM for 4–8 hours at 37 °C. Cells were then replated onto glass coverslips in supplemented DMEM, incubated for 2 hr at 37 °C, and then moved to 30 °C incubation overnight.

#### Histology

##### Whole Mount Histology:

Tissues were fixed in 4% paraformaldehyde (PFA) in PBS for 5 hr on a rocker and subsequently stored in PBS at 4°C until use. For immunofluorescence, samples were then washed with PBST (Triton X-100, 0.1%) 3 times and blocked for 1 hr in 10% normal goat serum (NGS) in PBST and antibody solution containing anti-HNK1 polyclonal antibody (1:100) (1C10 deposited by Halfter, WM Developmental Studies Hybridoma Bank) and anti-calretinin (1:100) (clone 3k22 #ZRB5054, Sigma) was applied for 24 hr at 4°C in NGS. Samples were then washed with PBST (TritonX 0.1%) 3 times, and goat anti-rabbit IgG H&L (Alexa Fluor^®^ 488) (Abcam ab6702) and Goat Anti-mouse IgG H&L (Cy3 ^®^) (AbCam ab97035) secondary antibodies were added (1:500). Finally, the samples were washed 5 times in PBST, mounted in Vectashield (Vector Laboratories), and imaged with an AxioZoom V16 Zoom Microscope (Zeiss) or LSM 980 Confocal Microscope with Airyscan2 (Zeiss). For DAPI staining, tissues were stained in 1μg/mL DAPI in 1XPBS for 30 min, washed 3X in PBS, and staged for imaging. Images were processed using FIJI.

##### Immunofluorescence staining of HEK Cells:

HEK293T cells were transfected as described above. Cells were washed 3 times in PBS and fixed in 4% paraformaldehyde (PFA) in PBS for 10 min at room temperature. Coverslips were then incubated in blocking mixture containing 1% bovine serum albumin (BSA) and 10 mM glycine in PBS. Samples were then incubated in antibody solution containing anti-FLAG^®^M2 monoclonal antibody (Sigma F1804) (1:100) and HA tag Polyclonal Antibody (Proteintech ab51064–2-AP) (1:250) for 24 hr at 4 °C in 1% BSA/PBS. Samples were then washed with PBS 3 times, and secondaries antibody mix containing goat anti-rabbit IgG H&L (Alexa Fluor^®^ 488) (Abcam ab6702) (1:500) and Goat Anti-mouse IgG H&L (Cy3 ^®^) (AbCam ab97035) (1:500) was added. Finally, the samples were washed 3 times in PBS, mounted in Vectashield with DAPI (Vector Laboratories), and imaged with a LSM 980 Confocal Microscope with Airyscan2 (Zeiss). Images were processed using FIJI.

##### Thin Section:

Tissues from the indicated species were fixed in 4% paraformaldehyde (PFA) in PBS for approximately 5 hr on a rocker and dissected into small sections, washed with PBS 3 times, and incubated in 30% sucrose in PBS at 4 °C on ice. After embedding in OCT (optimal cutting temperature compound), samples were frozen and sectioned using a cryostat (Leica CM3050S) at 20–50 μm sections. After subsequent washes in PBST, samples were blocked for 1 hr in 5% normal goat serum (NGS) in PBST and antibody solution containing anti-HNK1 polyclonal antibody (1:100) (1C10 deposited by Halfter, WM Developmental Studies Hybridoma Bank) and anti-calretinin (1:100) (clone 3k22 #ZRB5054, Sigma) in PBS was applied for 24 hr at 4 °C. Samples were then washed with PBST (TritonX 0.1%) 3 times, and goat anti-rabbit IgG H&L (Alexa Fluor^®^ 488) (Abcam ab6702) and Goat Anti-mouse IgG H&L (Cy3 ^®^) (AbCam ab97035) (1:500) secondary antibodies were added (1:500). Finally, the samples were washed 5 times in PBST, mounted in Vectashield with DAPI (Vector Laboratories), and imaged with a LSM 980 Confocal Microscope with Airyscan2 (Zeiss). Images were processed using FIJI.

#### T1r functional assays

Sea robin T1r plasmids were purified (Qiagen Plasmid Mini Kit) and *in vitro* responses were measured using a luminescence cell-based assay^16,17^. HEK293T cells (from the Matsunami Laboratory, Duke University, USA) were transiently co-transfected with expression vectors of sea robin T1rs, a rat G-protein (Gα15i2) and a photoprotein (mt-apoclytin-II) using Lipofectamine 2000 (Invitrogen). Control cells were only transfected with plasmids for the rat G-protein and the photoprotein (without receptor plasmids). Two days after transfection, cells were seeded in 96-well black-walled CellBIND plates (Corning) and assayed on a FlexStation3 microplate reader (Molecular Devices), which applied ligands and measured the luminescence intensity over 110 seconds in each well. All ligand solutions of various concentrations were prepared in filtered HEPES solution (4-(2-hydroxyethyl)-1-piperazine ethanesulfonic acid) at pH 7.4 (sugars: 100 mM, n = 6; L-amino acids: 50 mM, n = 6; D-alanine, isonipecotic acid, N,N-dimethylglycine, and L-2-aminobutyric acid: 50 mM, n = 5; sarcosine: 50 mM, n = 3; betaine and 2-amino-2-norbornanecarboxylic acid: 12.5 mM, n = 5; no ligand buffer, n = 6). The ligand-evoked responses were quantified as the area under the curve (AUC) and expressed as relative light units (RLU).

#### Patch clamp electrophysiology

Patch clamp recordings were carried out at room temperature using a MultiClamp 700B amplifier (Axon Instruments) and digitized using a Digidata 1550B (Axon Instruments) interface and pClamp software (Axon Instruments). Whole-cell recording data were filtered at 1 kHz and sampled at 10 kHz. Pipette resistances were 3–5 MΩ. The standard extracellular solution contained (in mM): 140 NaCl, 5 KCl, 10 HEPES, 2 CaCl_2_, 2 MgCl_2_, pH 7.4. The intracellular solution contained (mM): 140 Cs^+^ methanesulfonate, 1 MgCl_2_, 5 NaCl, 10 CsEGTA, 10 HEPES, 10 sucrose, pH 7.2. Whole-cell recordings were used to assess mechanical sensation with a piezoelectric-driven (Physik Instrumente) fire-polished glass pipette (tip diameter ~1 μm). Mechanical steps 150ms in duration were applied in 1 μm increments were applied every 5 s while cells were voltage-clamped at −80 mV.

#### Nerve recording

Legs from sedated animals were transferred to a 10 cm petri dish containing 50 mL holding solution (430 mM NaCl, 10 mM KCl, 10 mM HEPES, 10 mM CaCl_2_, 50 mM MgCl_2_, 10 mM D-glucose, pH 7.6). Nerve recordings were performed using a borosilicate glass suction electrode shaped and polished to fit over the entire cut end of the leg nerve. A similar reference electrode was placed in the bath. Gap-free recordings were made with 10 kHz sampling at 10,000 x gain, and signals were high-pass filtered at 100 Hz and lowpass filtered at 1 kHz using a Warner DP-311A headstage and AC/DC amplifier (Warner), and digitized using a Digidata 1440A digitizer (Molecular Devices) using ClampEx software (Molecular Devices). Recordings were processed using Clampfit (Molecular Devices). For quantification of responses, the absolute value of the signal was processed using a lowpass 25 Hz gaussian filter, the baseline signal was subtracted, and the response amplitude was integrated over the response area. Transients from pipette-bath-contact were removed from figure traces and not quantified. For controls and stimuli where no response was observed, a similar response area was measured as for leg agonists. For experiments testing chemical sensitivity of proximal versus distal legs, chambers were drawn around each leg region using blunted syringes loaded with 5% mineral oil mixed with melted petroleum jelly to create water-tight boundaries, and chemicals were perfused into the indicated chamber.

#### Agonists

Chemicals screened are included in the Key Resources table. Insoluble chemicals were first reconstituted in DMSO, and then diluted in ND96 to 0.1%−1% final DMSO. Fish extract was prepared by blending 1g frozen zebrafish per 2mL of holding solution, followed by centrifugation at 3,000 x g for 15 minutes to remove debris.

#### Quantification and statistical analysis

Data were analyzed with Clampfit (Axon Instruments), Prism Graphpad and represented as mean ± SEM. N represented independent experiments for the number of cells/patches or behavioral trials. Data were considered significant if p < 0.05 using paired or unpaired two-tailed Student’s t tests, Wilcox test, or one- or two-way ANOVAs. All significance tests were justified considering the experimental design and we assumed normal distribution and variance, as is common for similar experiments. Sample sizes were chosen based on the number of independent experiments required for statistical significance and technical feasibility.

## Supplementary Material

2

3 Video S1. Sea robin digging behavior, Related to Figure 1.
Example of a digging sea robin (*P. carolinus*) “walking” around the tank and probing a mussel shell. Video in real time.

## Figures and Tables

**Table:** KEY RESOURCES TABLE

REAGENT or RESOURCE	SOURCE	IDENTIFIER
Antibodies		
anti-HNK1 polyclonal antibody	Developmental Studies Hybridoma Bank	Cat#1C10
anti-calretinin antibody	Sigma	clone 3k22 Cat#ZRB5054
goat anti-rabbit IgG H&L (Alexa Fluor^®^ 488)	AbCam	Cat#ab6702
goat anti-mouse IgG H&L (Cy3 ^®^)	AbCam	Cat#ab97035
anti-FLAG^®^M2 monoclonal antibody	Sigma	Cat#F1804
HA tag Polyclonal Antibody	Proteintech	Cat#ab51064–2-AP
anti-calretinin antibody	Sigma	clone 3k22 Cat#ZRB5054
goat anti-rabbit IgG H&L (Alexa Fluor^®^ 488)	AbCam	Cat#ab6702
goat anti-mouse IgG H&L (Cy3 ^®^)	AbCam	Cat#ab97035
anti-FLAG^®^M2 monoclonal antibody	Sigma	Cat#F1804
HA tag Polyclonal Antibody	Proteintech	Cat#ab51064-2-AP
Biological Samples		
Tissue samples from *Prionotus carolinus*	Marine Biological Laboratory	n/a
Tissue samples from *Prionotus evolans*	Marine Biological Laboratory	n/a
*Prionotus punctatus* museum specimen in 70% ethanol	Harvard Museum of Comparatize Zoology	MCZ:Ich:13467
*Bellator militaris* museum specimen in 70% ethanol	Harvard Museum of Comparatize Zoology	MCZ:Ich:169373
*Trigla lyra* museum specimen in 70% ethanol	Harvard Museum of Comparatize Zoology	MCZ:Ich:24403
*Bellator loxias* museum specimen in 70% ethanol	Harvard Museum of Comparatize Zoology	MCZ:Ich:30779
*Chelidonichthys lucernus* museum specimen in 70% ethanol	Harvard Museum of Comparatize Zoology	MCZ:Ich:3832
*Lepidotrigla argus* museum specimen in 70% ethanol	Harvard Museum of Comparatize Zoology	MCZ:Ich:38511
*Prionotus stearnsi* museum specimen in 70% ethanol	Harvard Museum of Comparatize Zoology	MCZ:Ich:57741
*Prionotus scitulus* museum specimen in 70% ethanol	Harvard Museum of Comparatize Zoology	MCZ:Ich:57742
*Prionotus paralatus* museum specimen in 70% ethanol	Harvard Museum of Comparatize Zoology	MCZ:Ich:57756
*Prionotus alatus* museum specimen in 70% ethanol	Harvard Museum of Comparatize Zoology	MCZ:Ich:57764
*Peristedion gracile* museum specimen in 70% ethanol	Harvard Museum of Comparatize Zoology	MCZ:Ich:64852
Chemicals, Peptides, and Recombinant Proteins		
betaine	Sigma-Aldrich	Cat#61962
dimethylglycine	Sigma-Aldrich	Cat#D1156
sarcosine	Sigma-Aldrich	Cat#131776
isonipecotic acid	Sigma-Aldrich	Cat#I18008
β-alanine	Sigma-Aldrich	Cat#146064
nipecotic acid	Sigma-Aldrich	Cat#211672
L-pipecolic acid	Sigma-Aldrich	Cat#P2519
γ-aminobutyric acid	Hello Bio	Cat#HB0882
L-2-aminobutyric acid	Sigma-Aldrich	Cat#A1879
trimethylamine hydrochloride	Sigma-Aldrich	Cat#T72761
serotonin hydrochloride	Sigma-Aldrich	Cat#H9523
tyramine	Sigma-Aldrich	Cat#T90344
L-carnitine hydrochloride	Sigma-Aldrich	Cat#C0283
taurine	Sigma-Aldrich	Cat#T0625
L-proline	Sigma-Aldrich	Cat#P0380
glycine	Sigma-Aldrich	Cat#410225
L-cysteine	Sigma-Aldrich	Cat#168149
L-alanine	Sigma-Aldrich	Cat#W381829
L-leucine	Sigma-Aldrich	Cat#L8000
L-tyrosine	Sigma-Aldrich	Cat#T3754
L-phenylalanine	Sigma-Aldrich	Cat#P2126
L-glutamine	Sigma-Aldrich	Cat#G3126
L-methionine	Sigma-Aldrich	Cat#M22060
L-aspartic acid	Sigma-Aldrich	Cat#A9256
L-histidine	Sigma-Aldrich	Cat#H8125
L-ornithine	Sigma-Aldrich	Cat#O2375
L-tryptophan	Sigma-Aldrich	Cat#T0254
L-arginine	Sigma-Aldrich	Cat#A5006
L-lysine	Sigma-Aldrich	Cat#L5501
L-glutamate	Sigma-Aldrich	Cat#G1251
L-serine	Sigma-Aldrich	Cat#S4500
D-alanine	Sigma-Aldrich	Cat#A7377
D-cysteine	Sigma-Aldrich	Cat#30095
D-2-aminobutyric acid	Sigma-Aldrich	Cat#116122
D-serine	Sigma-Aldrich	Cat#S4250
D-leucine	Sigma-Aldrich	Cat#855448
D-cycloserine	Sigma-Aldrich	Cat#C6880
D-(+)-trehalose dihydrate	Sigma-Aldrich	Cat#T9531
sucrose	Sigma-Aldrich	Cat#S7903
D-(+)-galactose	Sigma-Aldrich	Cat#G6404
D-(+)-glucose	Sigma-Aldrich	Cat#49152
eucalyptol	Sigma-Aldrich	Cat#C80601
(R)-(+)-limonene	Sigma-Aldrich	Cat#183164
polygodial	Cayman Chemical	Cat#14979
carvacrol	Sigma-Aldrich	Cat#W224502
nootkatone	Sigma-Aldrich	Cat#W316620
sodium acetate trihydrate	Sigma-Aldrich	Cat#236500
sodium butyrate	Sigma-Aldrich	Cat#B5887
sodium propionate	Sigma-Aldrich	Cat#P1880
chloroquine diphosphate	Sigma-Aldrich	Cat#C6628
denatonium benzoate	Sigma-Aldrich	Cat#D5765
benztropine mesylate	Sigma-Aldrich	Cat#SML0847
gaboxadol hydrochloride	Sigma-Aldrich	Cat#T101
α-(methylamino)isobutyric acid	Sigma-Aldrich	Cat#M2383
BCH	Tocris	Cat#5027
adenosine	Sigma-Aldrich	Cat#A9251
inosine	Sigma-Aldrich	Cat#I4125
acetylcholine chloride	Sigma-Aldrich	Cat#A6625
isoamyl acetate	Sigma-Aldrich	Cat#W205532
choline chloride	Sigma-Aldrich	Cat#C7527
Yoda1	Selleck	Cat#S6678
neurotrophin-3	Sigma-Aldrich	Cat#SRP6007
collagenase IV	Worthington	Cat#LS004186
TSA plus Fluorescein	Perkin Elmer	Cat#NEL741001KT
TSA plus Cy3	Perkin Elmer	Cat#NEL744001KT
TSA plus Cy5	Perkin Elmer	Cat#NEL745001KT
Deposited Data		
RNA-seq reads	This study	**BioProject:** PRJNA1141128 : Evolution of novel sensory organs in fish with legs. Accession #SRR30014079-SRR30014098
Experimental Models: Cell Lines		
HEK293 cells	ATCC	Cat#CRL-1573
Experimental Models: Organisms/Strains		
sea robin: *Prionotus carolinus*: wild-type	Marine Biological Laboratory	
sea robin: *Prionotus evolans*: wild-type	Marine Biological Laboratory	
sea robin: *Prionotus scitulus*: wild-type	Gulf Marine Specimens	
sea robin: *Prionotus tribulus*: wild-type	Gulf Marine Specimens	
Oligonucleotides		
5’- GATTACGCCAAGCTTGGCAGTGGTATTCATTTGTGGCCTCAGG	Integrated DNA Technologies	*t1r1* 5’ RACE primer
5’- GATTACGCCAAGCTTCATGATCTTGCTTCTCTGTCTCTGCTGG	Integrated DNA Technologies	*t1r2* 3’ RACE primer
5’- GATTACGCCAAGCTTTTAGTTCCCCGGCTCTGGCTGTGGCTGT	Integrated DNA Technologies	*t1r3* 5’ RACE primer
Recombinant DNA		
Plasmid: pUNIV-HA-T1r1	Genscript	n/a
Plasmid: pUNIV-HA-T1r2	Genscript	n/a
Plasmid: pUNIV-FLAG-T1r3	Genscript	n/a
Plasmid: pEAK10-HA-T1r1	This study	n/a
Plasmid: pEAK10-HA-T1r2	This study	n/a
Plasmid: pEAK10-FLAG-T1r3	This study	n/a
Software and Algorithms		
Trinity v.2.4.0	Grabherr et al., 2011 ^26^.	
Diamond v.2.0.9	Buchfink et al., 2015 ^28^.	
Kallisto	Bray et al., 2016 ^27^.	
ImageJ2	NIH	
Zen	Zeiss	
Metamorph	BioVision Technologies	
pClamp v.11.3	Axon Instruments	
ClampFit v.11.3	Axon Instruments	
Prism v.10	GraphPad	

## Highlights:

- Sea robins are walking fish that use novel leg-like appendages as sensory organs
- A small clade of specialists elaborated sensory legs for locating buried prey
- Sensation is mediated by mechanosensitive neurons and epithelial taste receptors
- Leg papillae mediate sensory specialization across species and development
