# Supplementary material for: Evolution of novel sensory organs in fish with legs
Source: Curr Biol. Author manuscript; Available in PMC 2025 Jun 5. (PMC11552235; doi:10.1016/j.cub.2024.08.014)
Supplement: 2 [file NIHMS2019933-supplement-2.pdf]

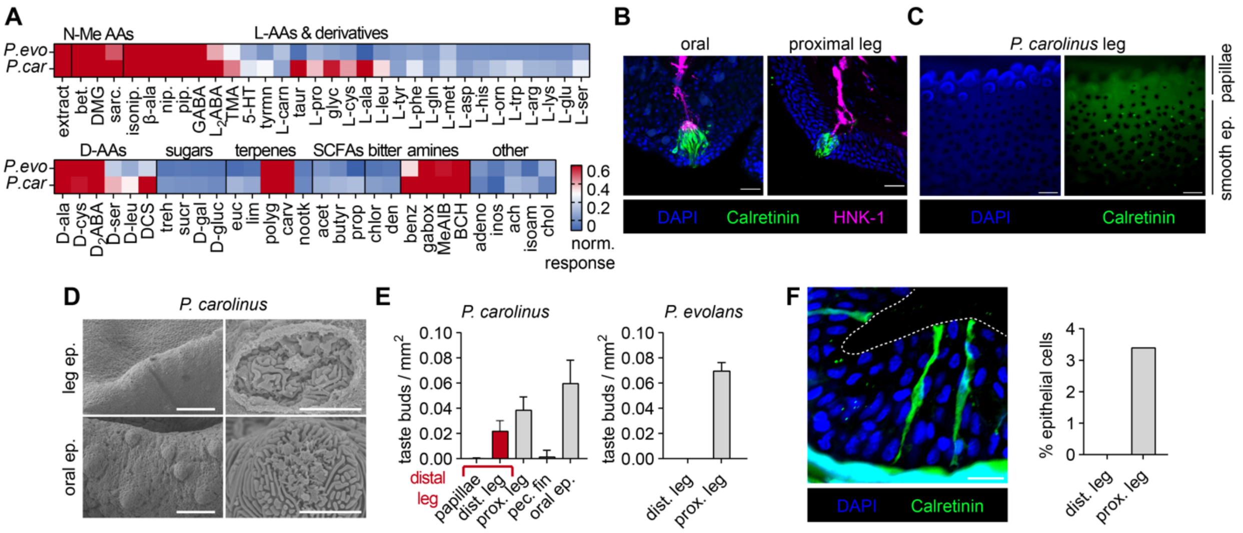

Figure S1. Sensory profiling of sea robin legs, Related to Figure 1 and Figure 2.
(**A**) Chemosensory responses from digging *P. carolinus* and non-digging *P. evolans* legs. Only *P. carolinus* were sensitive to appetitive L-amino acids. Heatmap of relative responses from > 6 legs. (**B**) Proximal legs of *P. carolinus* express calretinin in spindle-shaped cells that resemble taste buds which are commonly found in fish skin(*15*). Scale bars = 20 µm (*left*) and 200 µm (*right*). (**C-E)** These structures were absent from papillae as analyzed by immunofluorescence and scanning electron microscopy. SEM scale bars = 100 µm (*left*) and 5 µm (*right*). Data represented as mean ± s.e.m. (**F**) Calretinin-positive cells resembling solitary chemosensory cells were sparsely distributed throughout the skin of the proximal leg but absent from papillae of *P. carolinus*. Scale bar = 10 µm.

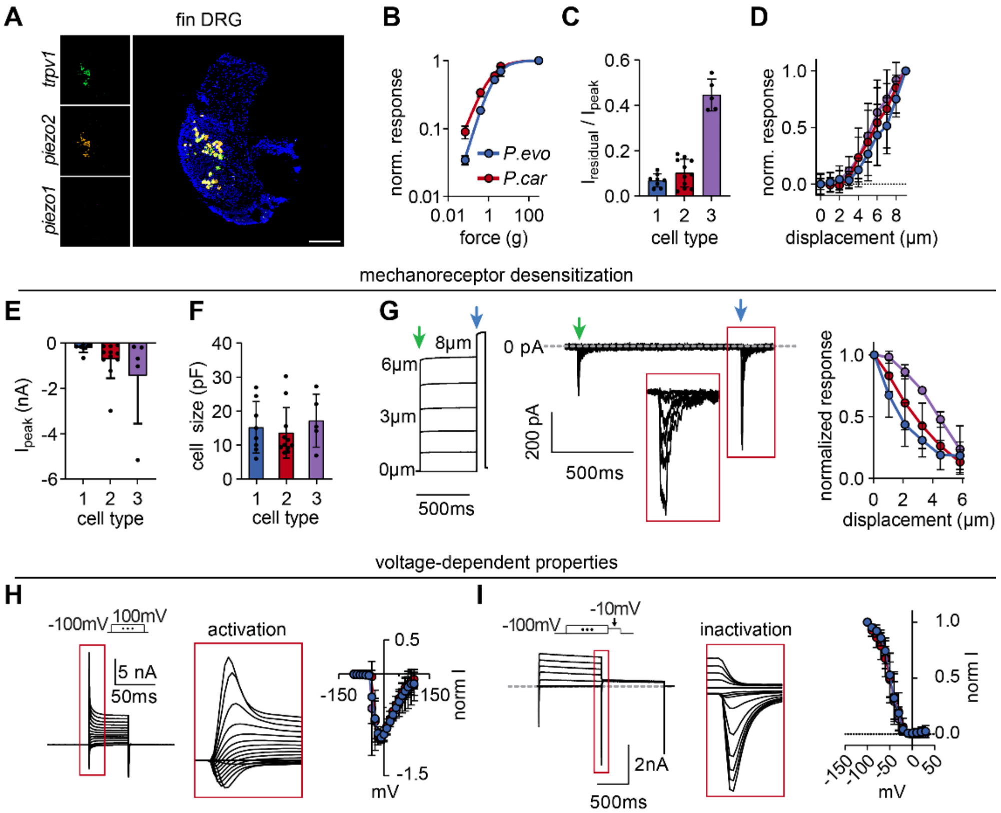

Figure S2. Mechanosensory properties of DRG neurons, Related to Figure 2 and Figure 3. (**A**) Fin-specific spinal ganglia contained *trpv1*-positive sensory neurons (green) that expressed the mechanoreceptor *piezo2* (orange) but not *piezo1* (pink, DAPI in blue, scale bar = 200 µm). Representative images of 3 animals per species. (**B**) Force-response curve using filament stiffness showed a higher sensitivity at low force for digging species *P. carolinus* compared with *P. evolans* (n > 5 stimulus-induced responses in recordings from 3 different legs). (**C**) Desensitization kinetics as measured by "residual" current at the end of the mechanical stimulus over peak stimulus-induced currents. Responsive cells could be segregated into three distinct mechanosensitive neuron populations (*n* = 26, 8 type 1 with fast desensitization, 11 type 2 with intermediate desensitization, and 5 type 3 with slow desensitization). (**D**) Mechanosensitive neuron populations exhibited similar thresholds for mechanosensitive responses (n = 8 type 1 in blue, 11 type 2 in red, 5 type 3 in purple). (**E**) Peak mechanosensitive currents in three mechanosensitive neuron subtypes (*n* = 8 type 1, 11 type 2, 5 type 3). (**F**) Capacitance of mechanosensitive neuron subtypes indicating similar approximate cell size (*n* = 8 type 1, 11 type 2, 5 type 3). (**G**) Desensitization of mechanically-induced currents in response to increasing long duration displacement was similar across subtypes (*n* = 8 type 1, 11 type 2, 5 type 3). (**H-I**), Voltage-dependent activation and inactivation of inward currents was similar across mechanosensitive neuron subtypes (n = 7 type 1, 10 type 2, 3 type 3). Data in **(B-I)** represented as mean ± s.e.m.

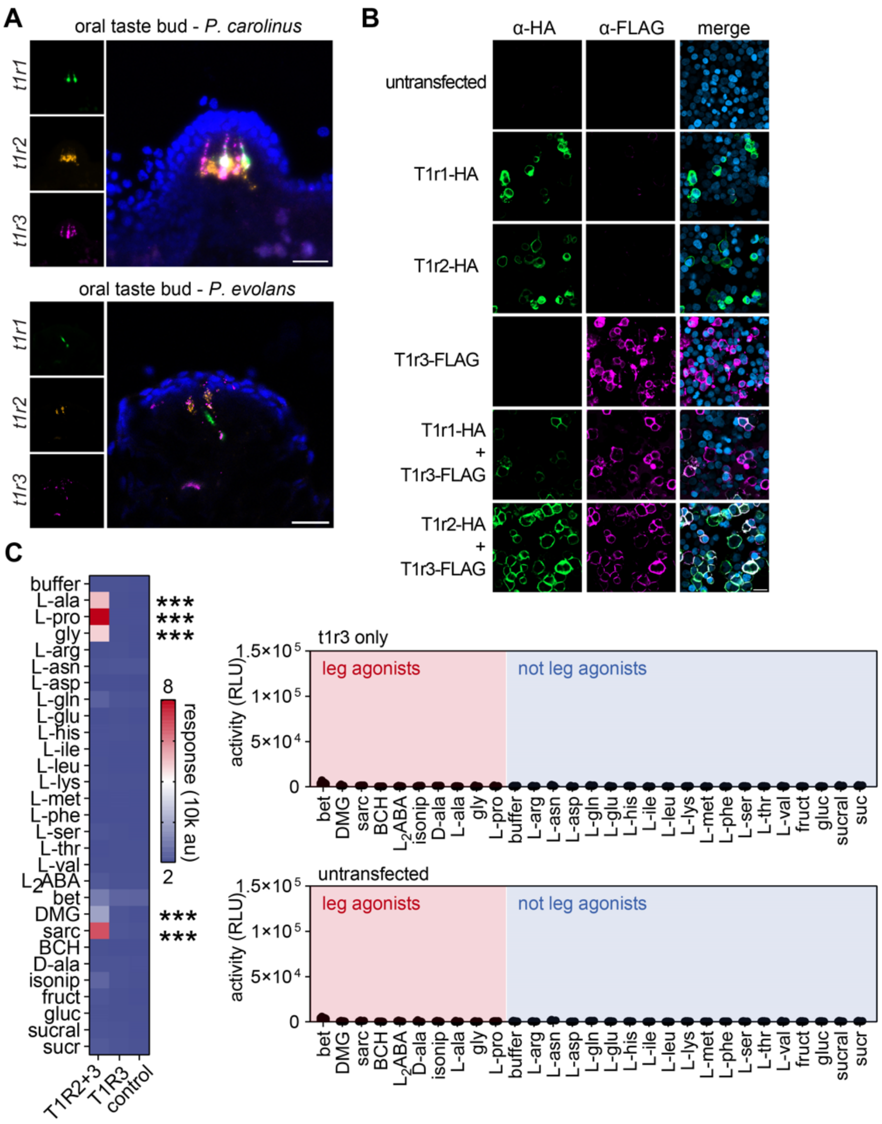

Figure S3. Taste receptor expression and function, Related to Figure 4.
(**A**) *t1r1*, *2*, and *3* were expressed in oral taste buds of *P. carolinus* and *P. evolans*, visualized by *in situ* hybridization (scale bars = 25 µm, images representative of 3 animals per species). (**B**) Epitope-tagged and codon optimized taste receptors were robustly expressed and trafficked to the plasma membrane in transfected HEK293T cells, as visualized by immunofluorescence microscopy. Scale bar = 25 µm. (**C**) Control HEK293T cells (lacking T1r receptors) and cells expressing only T1r3 did not respond to L-amino acids or other molecules like T1r2-T1r3 heterodimers (n = 6, two-way ANOVA with Tukey's post-hoc test. RLU = Relative Light Units. *** indicates $p < 0.0001$. Statistical significance indicates comparison of T1r2 + T1r3 co-expression to T1r3 only and control cells. Data represented as the mean ± s.e.m.

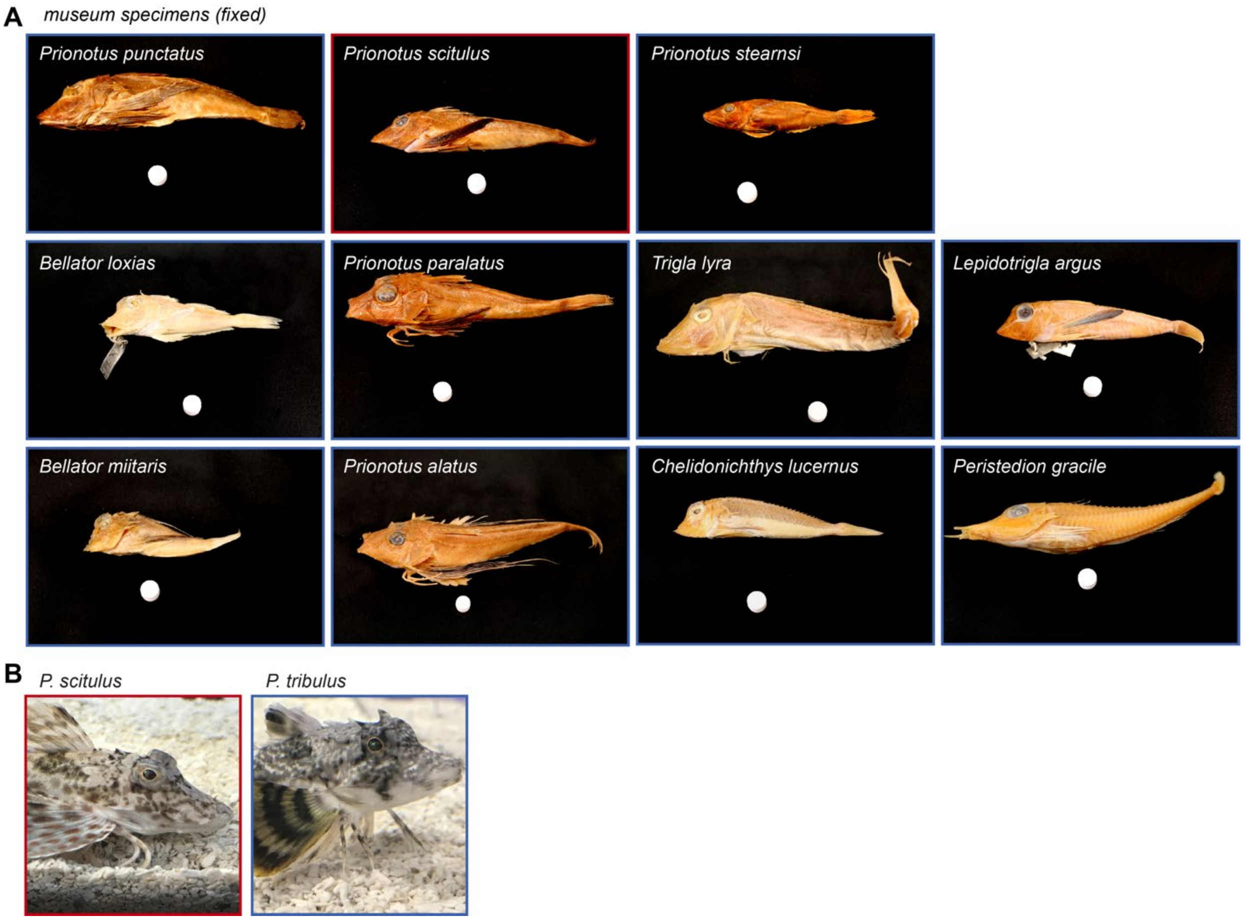

Figure S4. Comparative morphology, Related to Figure 5.
(**A**) Museum specimens used for leg morphological analysis. Scale dot = 1 cm. (**B**) Lateral views of the wild-caught *P. scitulus*, which had leg papillae, and *P. tribulus*, which lacked papillae, used in physiological and behavioral experiments. Red outline = papillae, blue outline = no papillae.
